# Supplementary material for: RAMCOM: A qualitative study of clinicians’ viewpoints on a tool for communication with Muslim patients considering fasting during Ramadan
Source: PLoS One. 2020 Feb 13;15(2):e0228888. doi: 10.1371/journal.pone.0228888 (PMC7017985; doi:10.1371/journal.pone.0228888)
Supplement: Attachment — Submitted filename: RAMCOM Manuscript, revised.docx [file pone.0228888.s001.docx]

**RAMCOM: A tool for communication with Muslim patients considering fasting during Ramadan**

**First and Corresponding Author:**

**Mohamed Ezzat Khamis Amin, BSc Pharm Sci, MS, PhD**

Associate Professor

Beirut Arab University Faculty of Pharmacy

P.O. Box: 11 5020 Beirut, Lebanon
Phone: +961 1 300110 Ext: 2792

[m.amin@bau.edu.lb](mailto:m.amin@bau.edu.lb)

**Second Author:**

**Ahmed Abdelmageed, PharmD**

Associate Professor

College of Pharmacy, Natural and Health Sciences

Manchester University

10627 Diebold Rd.

Fort Wayne, Indiana, USA 46845

260-470-2676

[aabdelmageed@manchester.edu](mailto:aabdelmageed@manchester.edu)

**Abstract**

**Objectives:** Fasting during the month of Ramadan is a basic pillar of Islam. While patients may be religiously exempted from fasting, literature indicates that the majority decide to fast. Caring for millions of Muslim patients who decide to fast during Ramadan can be challenging for clinicians around the globe. This study proposes a communication tool, RAMCOM, that aims to assist clinicians in communicating with Muslim patients considering fasting during Ramadan. It also gathers clinicians’ perspectives on implementability and dissemination of the proposed tool.

**Methods:** Semi-structured interviews were conducted with a purposeful sample of clinicians in Egypt (11) and the US (10). Clinicians were purposefully sampled to assure variance in age, gender, time in practice, specialty, and religious background. Directed content analysis was conducted and emerging data were mapped to constructs within the theory of planned behavior. Iterative sampling and analysis continued until saturation was reached.

**Results:** In total, 21 clinicians were interviewed. The tool was iteratively revised according to clinicians’ comments on format, content, language and usability. Factors contributing to using RAMCOM included perception of tool (need for use, perceived burden of use), perceived norm (perceived patient expectations), and ability to use tool (time, frequency of seeing patients, knowledge of Ramadan and Islam). Practice environment factors that impact the use of RAMCOM include education, early reminders, colored laminated copies, communication training, involvement of support staff, and patient education.

**Conclusion:** Clinicians provided valuable perceptions on the implementability and use of RAMCOM, a new communication tool designed to assist in caring for Muslim patients during Ramadan. These perceptions should be considered by different stakeholders to facilitate goal-concordant care for Muslim patients considering fasting.

# Introduction

Fasting during the month of Ramadan, the ninth month of the lunar calendar, is a basic pillar of Islam. Observing this month is an extremely important ritual to many Muslims who make nearly a quarter of the world’s population [1]. From dawn to sunset in Ramadan, Muslims who are able, are expected to completely abstain from eating, drinking, and sexual intercourse. The usual practice is to have a pre-fast meal (Suhour) before dawn and a post-fast meal after sunset (Iftar). In addition to the five daily prayers, some do nonobligatory night prayers known as Taraweeh, which contribute to their daily exercise.

According to mainstream Islamic scholars, fasting is incompatible with using oral dosage forms, which nullify the fast. Patients, however, can take oral dosage forms before dawn and after sunset when eating and drinking is allowed without compromising the daily fast. In consonance with Islamic teachings, fasting is not meant to create hardship on worshipers. Muslims who are temporarily sick, traveling, pregnant, breastfeeding or menstruating, are exempt from fasting, but they must later make up any missed days upon recovery, return, or after weaning. Patients with chronic conditions whose health may be negatively influenced by a change in medication use or diet associated with fasting are exempt. In fact, they should not be participating in the ritual to prevent self-harm, which is prohibited according to Islamic teachings. While Muslim patients need not apply for an exemption from any religious authority, literature indicates that some Muslim patients like to consult imams, religious scholars and clinicians when trying to identify whether they have a religious exemption from fasting or not and which dosage forms may or may not nullify it [2]. Earlier work indicates that patients may differ in their perceptions on dosage forms they can use while fasting. For example, some patients think that inhalers, eye drops, suppositories, injections nullify the fast while others do not [3,4]. Although patients may be aware of their religious exemption from fasting, studies demonstrated that the majority of Muslim patients choose to fast rather than take the exemption [5]. In some cases, patients may adjust their therapeutic regimens without consulting a clinician resulting in patients omitting doses, taking medications at closer intervals, or at times stopping one or more medications altogether, which could lead to increased risk for patients.

A number of studies, the majority of which were done in the past decade and in non-Muslim countries, identified communication gaps between clinicians and patients in relation to fasting during Ramadan [6,7,16,8–15]. Clinicians and patients may not have a conversation that tackles the issue to begin with. At times, those conversations may occur, but these often do not cover important issues such as adjusting medications, risks of fasting, indications to break fasting, meal plans and exercise. While some guidance has been provided to clinicians when communicating with patients about Ramadan, [17,18] there is no structured published tool for clinicians to use when communicating with patients who are considering fasting during Ramadan.

This manuscript is part of a larger study dataset examining clinician-patient communication in relation to Ramadan. The first manuscript used social cognitive theory as a guiding framework to explore factors affecting counseling provided to Muslim patients considering fasting Ramadan from a clinician’s perspective [19]. This second manuscript complements earlier findings by proposing a communication tool, that aims to assist clinicians in communicating with Muslim patients considering fasting during Ramadan. It also gathers clinicians’ perspectives on implementability and dissemination of the proposed tool.

# Methods

## *Construction of initial draft of RAMCOM*

This tool was designed with the goal of providing clinicians with a resource that would assist them in providing patient-centered care to Muslim patients who are considering fasting during Ramadan. To achieve this, the proposed tool follows the seven essential sets of communication tasks described in the Kalamazoo consensus statement: (1) build the doctor-patient relationship; (2) open the discussion; (3) gather information; (4) understand the patient's perspective; (5) share information; (6) reach agreement on problems and plans; and (7) provide closure.[20] Moreover, specific components of RAMCOM address issues that have been identified from research related to clinician patient communication about Ramadan. Earlier research indicates that patients generally preferred more autonomous decision making when it comes to fasting during Ramadan. Accordingly, the tool follows a model of shared decision making where both the patient and the clinician contribute to the decision. It also helps clinicians show some awareness of the basic tenets of Ramadan fasting and its significance early in the encounter. It encourages clinicians to demonstrate empathy whenever an opportunity is provided, which has been shown to improve the quality of communication and care, especially in cross cultural communication [21].

The tool encourages clinicians to ask patients whether they think the dosage form(s) they are currently using nullifies their fast or not. If a patient thinks the dosage form nullifies their fast, then it cannot be taken from dawn to sunset while fasting, but if possible, it might be moved to the period from sunset to dawn. In case the patient decides to fast, the tool encourages clinicians to be explicit about potential risks of fasting, use of medications, indications to break fasting, diet and meal times, and exercise. The use of written materials when possible is also emphasized.

Given that the tool was introduced to Arabic speaking clinicians in Egypt along with English speaking clinicians in the United Stated, English and Arabic versions of RAMCOM were prepared by the first author. To ensure consistency among the Arabic and English versions of the tool, an independent physician who is fluent in both languages was recruited to verify the accuracy of the translation.

## *Participants and recruitment*

Ethical approval for the study was obtained from the Manchester University Institutional Review Board with written informed consent from participants (Study Number: 1617FW12). Recruitment and interviews of clinicians took place from May to September 2017. In 2017, Ramadan (Hijri year 1438) started on May 26^th^ and ended on June 24^th^. Details of sample selection, recruitment and strategies addressing trustworthiness as defined by Lincoln and Guba [22] have been described in the first manuscript published from this project [19]. Ongoing preliminary data analysis has been conducted during data collection. The study did not have a target sample size; rather it aimed to recruit participants until saturation of key themes occurred. More specifically, a strategy involving a priori thematic saturation, which relates to the degree to which identified codes or themes are exemplified in the data, was followed [23]. The process of iterative sampling and analysis continued throughout the study.

## *Interview method and research instrument*

Interviews were conducted in-person (11) and over the phone (9). All interviews were conducted by the first author with exception of one, which was conducted by the second author. All interviews were recorded using an audio recorder with participant permission. Recordings were transcribed verbatim following each interview by native speakers in the language used to conduct the interview. Field notes were created by the first author during interviews. These notes were used to supplement interview data and helped the research team in producing meaning and understanding of the issue being studied.

The most recent version of the tool was sent electronically to each clinician ahead of each interview. The interview began in an open-ended manner, getting participants to talk about their typical encounter with Muslim patients before Ramadan. Open-ended, neutral questions were carefully constructed to reduce the chance of eliciting socially desirable responses. The interviewer asked participants to reflect on any factors that would facilitate or prohibit them from using the tool. Items derived from the GUIDE-IT tool framed questions of the initial interview guide that addressed the tool’s implementability [24]. Participants were also asked about how they think the tool should be introduced to clinicians and how to have it available at their practice sites. Appropriate probes were used throughout the interviews when the interviewer wanted to obtain a more in-depth response. To establish face validity, the interview guide was pre-tested with an Egyptian physician. Only minor changes to how the researcher phrased the questions resulted from pre-testing.

## *Data analysis*

The main unit of analysis was the individual narratives of interviewed clinicians. The coding process began with the identification of passages from the transcribed interviews which fit the constructs, yielding to the reported themes. The first author started familiarizing himself with the data through reading and re-reading transcripts, noting the participants’ use of particular words or thought patterns emerging from the data. This was followed by discussions among the two authors. The template analysis style where the text was organized and presented according to preexisting categories was used.[25] Directed content analysis[26] was performed based on components of the GUIDE-IT tool[24] including format, content and language and the theory of planned behavior (TPB)[27]. According to the TPB, attitudes (clinicians’ attitude towards RAMCOM), subjective norms (perception of whether patients approve using RAMCOM) and perceived behavioral control (perception of the difficulty of using RAMCOM in practice) determine the individual’s behavioral intention (clinicians’ plan to use RAMCOM in caring for patients) and consequently determine the likelihood of the individual carrying out that specific behavior.

# Results

In total, 20 physicians and one ambulatory care pharmacist were interviewed. Participants were practicing in Egypt (11) and the US (10); thirteen were male. Experience as practitioner ranged from 11 months to 38 years. Nine participants were non-Muslims including two non-Muslim clinicians from Egypt and seven from the US. Self-identified clinicians’ areas of practice included Family Medicine (6), Internal Medicine (3), Diabetology (3), Tropical Medicine & Hygiene (2), Pulmonology (1), Cardiology (1), Gastroenterology (1), Ophthalmology (1), Nephrology (1), Psychiatry (1), and Obstetrics & Gynecology (1). Participants’ average age was 45 and ranged from 28 to 68 years. Two participants did not read the tool prior to the interview and were unable to provide comments on its content and usability. They did, however, provide comments on dissemination and other issues related to barriers and facilitators for implementation. The mean duration for those interviews was 30 minutes (range 21– 44).

## *Perceptions of RAMCOM*

### *Content, Format, Style, Length and language*

Several clinicians preferred the tool to be only one page long. Accordingly, the first draft of the tool, which was nearly 2-3 pages long was shortened to one page. The final version of RAMCOM is shown in figure 1.

There was considerable variation in preference for hard copy versus electronic versions of the tool with some clinicians preferring to have both versions available. Specifically, clinicians cited the possibility of giving a colored laminated copy of the tool to the patient during discussions to build rapport as reasons for preferring a hard copy version of the tool. Seeing the entire process on a single piece of paper rather than having to go through multiple screens in an electronic version was an additional reason for preference. Others clinicians, however, preferred having an electronic version citing the ability to pull it on a smart phone anywhere and at anytime as compared to a hard copy tool that could be set aside and lost. The ability to access other information simultaneously through pulling another screen was viewed as an additional advantage. Egyptian clinicians, however, cited the inaccessibility of technology in many settings as a significant barrier.

While most clinicians felt that they would prefer the flowchart format given the way it is organized and its similarity to other algorithms they use, one clinician felt the bullet point format provides better flexibility that would help him better adapt the interview to the patient.

Participants’ suggestions about the language used were minimal and were addressed as the study proceeded. A clinician in Egypt indicated that he particularly appreciated that the Arabic version of the tool was prepared in the Egyptian dialect, which would help clinicians convey the messages clearly to patients without the burden of translating it from English or classical Arabic. Several clinicians indicated they preferred the tool in the form of scripted questions and phrases to say rather than directions of what should be done. The tool was revised in accordance with this format.

*Value of tool*

Several US clinicians felt that parts of the tool, such as the one where they ask patients on whether they feel a dosage form would nullify fasting or not, were informative in themselves. While some clinicians felt that the tool would be useful for them to utilize, others, showing signs of high self-efficacy for caring for patients in relation to Ramadan, felt they don’t need to use it because dealing with patients who observe Ramadan on a regular basis has given them the necessary skills to drive such conversation. Negative prior perceptions about other regimented tools and policies regarding their use, especially with US clinicians who frequently deal with them, were cited as a reason why clinicians may resist using RAMCOM.

*Perceived norm*

A key issue for clinicians was on how to proceed with the first step of the tool, which involves identifying the patient as a Muslim. While Egyptian clinicians, including non-Muslim ones, did not cite this as a problem, perceptions of US clinicians were mixed. Some US clinicians felt that they were comfortable addressing this, especially if the patient has a Muslim name or wear hijab. Others felt that the norm in the US makes it uncomfortable both for clinicians and patients to bring this up.

Three Egyptian clinicians argued that they would reserve the concordant approach of using the tool for patients who would be a good fit for this kind of shared decision making. When probed about the criteria they use to identify such patients, they described patients with higher degree of education. Physician N explains how using a concordant approach with patients of lower education would defy patients’ expectations for his role and would result in patients having doubts about his ability to manage their condition:

“A patient may not like this way (of communication). Some patients like to have the doctor to be their superior *‘Do this and take that treatment. Don’t discuss it with me’*. That’s the majority, especially those, I am sorry to say, of low socioeconomic status. If I do this (use the tool), they would say *‘the doctor is not good, he keeps on asking us questions. What kind of a doctor would keep on asking me questions? Why am I coming to see him then’*…. and they would leave me”

*Ability to use tool*

Awareness about Islam and Ramadan was a key factor impacting the clinicians’ ability to use the tool. Non-Muslim clinicians in the US, especially, ones who had limited to no prior knowing exposure to Islam and Muslims felt that education about these topics would help them in using the tool more effectively to address patients’ needs. Time spent with patients was cited as another significant factor that would impact the clinician’s ability to use the tool. As expected, clinicians who feel that they have time to address the issue of fasting thoroughly were more confident about their ability to use RAMCOM as a guiding tool.

### *Clinicians’ suggestions for dissemination and implementation*

Clinicians recommended several strategies for disseminating the tool. Those included covering the tool in debriefings and one to one clinician visits and introducing it at national and international conferences. Younger clinicians suggested incorporating the tool in cell-phone applications and adding it to electronic evidence-based clinical decision support resources such as UpToDate. Involving different stakeholders including medical societies, universities and government agencies was cited as a strategy that would help in disseminating the tool and sustaining its use.

Training and educating clinicians and their support staff on issues related to Ramadan, Islam and communication with patients in general were key recommendations for facilitating the use of RAMCOM in practice. The use of case studies in training was specifically cited as a potentially helpful strategy. Should they be available, coupling RAMCOM with clinical guidelines for different conditions was also viewed as something that would help clinicians utilize it. Given the fact that the lunar calendar does not correspond with the ubiquitously used Gregorian calendar, Ramadan occurs around 11 days earlier every year. Accordingly, clinicians felt that sending annual reminders about the tool and its use weeks to months before Ramadan could be a useful strategy for institutions. Identifying and making outreach efforts to Muslim patients in the community and patients served by clinicians’ institutions was viewed as an additional strategy that could help patients bring the topic to clinicians, which would enable them to identify patients to go over the tool with.

[Figure 1 near here].

# Discussion

Building on the literature on implementability, [24] this study describes the process of developing RAMCOM, a communication tool designed to assist clinicians in communicating with patients about fasting during Ramadan. As hypothesized, examining constructs related to perceived value, subjective norm and perceived ability to use the tool provided useful insights in relation to the tool’s implementability. In addition, the study provided guidance from clinicians in different settings on useful strategies for disseminating the tool.

Findings from this study have implications when considering the specific context in which the tool will be used. As indicated by clinicians, preferences can vary considerably not just in different countries and health systems, but even within a single institution. For instance, while opportunities for integration of the tool within electronic resources should be explored, it is important to provide clinicians with preference for more traditional modes an opportunity to be trained on and apply the tool. Likewise, dissemination attempts should be adapted to address audience in different specialties and geographic places rather than solely focus on clinicians dealing with diabetic patients.

Moreover, for a better use of the tool, patients should be better empowered to ask questions. Training patients to take the initiative and start a conversation with clinicians on the topic would make it easier for clinicians to apply the tool. Literature, however, indicated that patients are reluctant to bring this up for a number of reasons, including prior experiences with clinicians and patient concerns about straining their relationship with clinicians and perceptions of Muslims among clinicians with little background of dealing with this group [11,28]. One way of empowering patients to address this issue is to provide them with a patient version of the RAMCOM tool that mirrors the clinician version of the tool [15]. This could increase the chances of having a more goal-concordant care for this class of patients.

Respecting the autonomy of patients with different backgrounds is a key principle of biomedical ethics. Some clinicians in this study, however, indicated they were unlikely to use the tool as part of a concordant approach with low socioeconomic patients. These results corroborate the findings of a great deal of the previous work in clinician patient communication, which indicates that the doctors commonly misperceive the desire and need for information of patients of low socioeconomic status along with their ability to contribute to making decisions in their care process. Moe specifically, patients with low socioeconomic status receive “less positive socio-emotional utterances and a more directive and less participatory consulting style, characterized by significantly less information giving, less directions and less socio-emotional and partnership building utterances from their doctor” [29]. An alternative approach would involve assessing preferences for patients with all backgrounds, rather than only ones with low socioeconomic status, on whether they wished to participate in decisions or whether they wish to delegate them to clinicians. This should be done while noting that a patient’s role preference might change as the visit proceeds. [30]

It is important to note that, ideally, such tool should be viewed as one of several components in interventions addressing this issue. To be successful, a comprehensive intervention would involve all aspects impacting the patient care process in relation to Ramadan. Institutional support is extremely important in overcoming organizational barriers in implementing initiatives aiming at caring for Muslim patients during Ramadan [31]. Institutional support would also be key in providing clinicians with proper training addressing Muslim patients, Ramadan, and other background information that would help in applying the tool. At this point in time, clinical guidelines for transitioning patients safely in and out of Ramadan are generally lacking. There are, however, some intensive resources for therapeutic guidance on caring for diabetic patients produced by the Diabetes and Ramadan International Alliance (DaR) and other collaborators such as Diabetes Canada [32]. Those guidelines can be used along with RAMCOM when training clinicians to assist them in the care process. Sessions introducing the patient’s perspective could helpful, especially in cases where clinicians are under the false assumption that patients are satisfied with the communication process when in fact, they may not be. Finally, RAMCOM should be introduced to clinicians as a guiding tool that would help them in the care process rather than an additional tool that is imposed on them, to avoid triggering the resistance clinicians have reported with regimented tools previously introduced at their practice sites.

**Strengths and limitations**

This study sample included clinicians from countries with different proportions of Muslim populations and different health systems. This in addition to the considerable variation in characteristics and perspectives of participating clinicians brought richness to generated data addressing RAMCOM, its implementability and dissemination. On the other hand, it is possible that some clinicians refrained from giving negative criticism about the communication tool knowing that the authors have designed it. It is likely that discussions following wider dissemination of the tool would further help in refining it.

# Conclusions

Clinicians provided valuable perceptions on the implementability of RAMCOM and its use in caring for Muslim patients. To facilitate better care for Muslim patients considering fasting, these perceptions should be considered by different stakeholders. Further studies should explore the value of RAMCOM in future interventions addressing this communication gap.

**Acknowledgments**

We are very indebted to colleagues who supported this project in Egypt and the US. We are particularly thankful for Dr. Betty Chewning for valuable methodological advice, Dr. Ahmed Osman, Dr. Mohammad Shoukry Newegy, Dr. Brian Henriksen, and Dr. Joshua Kline, for technical support in collection of data, Mrs. Eman ElNaggar, Ms. Marwa Farhat for transcription of data. We would also like to thank all clinicians who took the time to participate and provide valuable perceptions about the tool.

**REFERENCES**

1.	Pew Research Center’s Forum on Religion & Public Life. The Future of the Global Muslim Population : Projections for 2010-2030 [Internet]. Washington, District of Columbia: Pew Research Center Forum on Religion and Public Life; 2011. [cited 2019 Nov 1]. Available from: https://www.pewforum.org/2011/01/27/the-future-of-the-global-muslim-population/

2.	Ilkilic I, Ertin H. Ethical conflicts in the treatment of fasting Muslim patients with diabetes during Ramadan. Med Heal Care Philos. 2017;20: 561–570. doi:10.1007/s11019-017-9777-y

3.	Kumar N, Dherani M, Jivan S. Ramadan and eye-drops: perspective of Muslims in the UK. Br J Ophthalmol. 2009;93: 551–552. doi:10.1136/bjo.2008.151001

4.	Ali R, Siddiqui H, Anjum Q, Lohar MI, Shaikh SS. Knowledge and perception of patients regarding medicine intake during Ramadan. J Coll Physicians Surg Pak. 2007;17: 112–113. Available: http://www.ncbi.nlm.nih.gov/pubmed/17288862

5.	Salti I, Benard E, Detournay B, Bianchi-Biscay M, Le Brigand C, Voinet C, et al. A population-based study of diabetes and its characteristics during the fasting month of Ramadan in 13 countries: results of the epidemiology of diabetes and Ramadan 1422/2001 (EPIDIAR) study. Diabetes Care. 2004;27: 2306–2311.

6.	Amin MEK, Chewning B. Pharmacist–patient communication about medication regimen adjustment during Ramadan. Int J Pharm Pract. 2016. doi:10.1111/ijpp.12282

7.	Savas E, Ozturk ZA, Tanriverdi D, Kepekci Y. Do Ramadan Fasting Restrictions Alter Eating Behaviours in Obese Women? J Reli Heal. 2012. doi:10.1007/s10943-012-9605-x

8.	Pinelli NR, Jaber LA. Practices of Arab American patients with type 2 diabetes mellitus during Ramadan. J Pharm Pr. 2011;24: 211–215. doi:10.1177/0897190010367432

9.	Peeters B, Mehuys E, Van Tongelen I, Van Bever E, Bultereys L, Avonts D, et al. Ramadan fasting and diabetes: An observational study among Turkish migrants in Belgium. Prim Care Diabetes. 2012; 293–296. doi:10.1016/j.pcd.2012.02.003

10.	Patel NR, Kennedy A, Blickem C, Rogers A, Reeves D, Chew-Graham C. Having diabetes and having to fast: a qualitative study of British Muslims with diabetes. Heal Expect. 2015;18: 1698–1708. doi:10.1111/hex.12163

11.	Mir G, Sheikh A. “Fasting and prayer don’t concern the doctors ... they don’t even know what it is”: communication, decision-making and perceived social relations of Pakistani Muslim patients with long-term illnesses. Ethn Heal. 2010;15: 327–342. doi:10.1080/13557851003624273

12.	Mygind A, Kristiansen M, Wittrup I, Norgaard LS. Patient perspectives on type 2 diabetes and medicine use during Ramadan among Pakistanis in Denmark. Int J Clin Pharm. 2013;35: 281–288. doi:10.1007/s11096-012-9716-1

13.	Zeeneldin AA, Gaber AA, Taha FM. Does fasting during Ramadan trigger non-adherence to oral hormonal therapy in breast cancer patients? J Egypt Natl Canc Inst. 2012;24: 133–137. doi:10.1016/j.jnci.2012.06.003

14.	Myers PR, Shoqirat N, Allen DH, Dardas LA. Patients with diabetes observing Ramadan: The experience of Muslims in the United States. Diabetes Res Clin Pract. 2019. doi:10.1016/j.diabres.2018.12.011

15.	Amin MEK, Abdelmageed A, Farhat MJ. Communicating with Clinicians on Fasting during Ramadan: The Patients’ Perspective. J Relig Health. 2019. doi:10.1007/s10943-019-00910-x

16.	Robinson T, Raisler J. “Each one is a doctor for herself”: Ramadan fasting among pregnant Muslim women in the United States. Ethn Dis. 2005;15: S1-99–103.

17.	Kristiansen M, Sheikh A. Pre-existing Health Concerns and Their Management in Ramadan and Mass Gatherings. In: Laher I, editor. Handbook of Healthcare in the Arab World. Springer, Cham; 2019. pp. 1–13. doi:https://doi.org/10.1007/978-3-319-74365-3_53-1

18.	Alomi Y. Update 2018- Drug Therapy during Holy Month of Ramadan. Riyadh, Saudi Arabia: Saudi Ministry of Health, General Adminsitration of Pharmaceutical care; 2018 May.

19.	Amin MEK, Abdelmageed A. Clinicians’ Perspectives on Caring for Muslim Patients Considering Fasting During Ramadan. J Relig Health. 2019. doi:10.1007/s10943-019-00820-y

20.	Makoul G. Essential elements of communication in medical encounters: the Kalamazoo consensus statement. Acad Med. 2001;76: 390–393.

21.	Lorié Á, Reinero DA, Phillips M, Zhang L, Riess H. Culture and nonverbal expressions of empathy in clinical settings: A systematic review. Patient Educ Couns. 2017. doi:10.1016/j.pec.2016.09.018

22.	Lincoln YS, Guba EG. Naturalist inquiry. Beverly Hills, CA: Sage Publications; 1985.

23.	Saunders B, Sim J, Kingstone T, Baker S, Waterfield J, Bartlam B, et al. Saturation in qualitative research: exploring its conceptualization and operationalization. Qual Quant. 2018. doi:10.1007/s11135-017-0574-8

24.	Kastner M, Estey E, Hayden L, Chatterjee A, Grudniewicz A, Graham ID, et al. The development of a guideline implementability tool (GUIDE-IT): a qualitative study of family physician perspectives. BMC Fam Pr. 2014;15: 19. doi:10.1186/1471-2296-15-19

25.	Miller WL, Crabtree BF. Qualitative analysis: how to begin making sense. Fam Pr Res J. 1994;14: 289–297.

26.	Hsieh HF, Shannon SE. Three approaches to qualitative content analysis. Qual Heal Res. 2005;15: 1277–1288. doi:10.1177/1049732305276687

27.	Ajzen I. The theory of planned behavior. In: Van Lange PAM, Kruglanski AW, Higgins ET, editors. Handbook of theories of social psychology (Vol 1). Thousand Oaks, CA: Sage Publications Ltd; 2012. pp. 438–459.

28.	Pathy R, Mills KE, Gazeley S, Ridgley A, Kiran T. Health is a spiritual thing: perspectives of health care professionals and female Somali and Bangladeshi women on the health impacts of fasting during Ramadan. Ethn Heal. 2011;16: 43–56. doi:10.1080/13557858.2010.523780

29.	Willems S, De Maesschalck S, Deveugele M, Derese A, De Maeseneer J. Socio-economic status of the patient and doctor-patient communication: does it make a difference? Patient Educ Couns. 2005;56: 139–146. doi:10.1016/j.pec.2004.02.011

30.	Chewning B, Bylund CL, Shah B, Arora NK, Gueguen JA, Makoul G. Patient preferences for shared decisions: a systematic review. Patient Educ Couns. 2012;86: 9–18. doi:10.1016/j.pec.2011.02.004

31.	Almansour HA, Chaar B, Saini B. Pharmacists’ perspectives about their role in care of patients with diabetes observing Ramadan. Research in Social and Administrative Pharmacy. 2016: 109–122. doi:10.1016/j.sapharm.2016.02.006

32.	Bajaj HS, Abouhassan T, Ahsan MR, Arnaout A, Hassanein M, Houlden RL, et al. Diabetes Canada Position Statement for People With Types 1 and 2 Diabetes Who Fast During Ramadan. Can J Diabetes. 2019. doi:10.1016/j.jcjd.2018.04.007
